# Supplementary material for: CELF RNA binding proteins promote axon regeneration in C. elegans and mammals through alternative splicing of Syntaxins
Source: eLife. 2016 Jun 2;5:e16072. doi: 10.7554/eLife.16072 (PMC4946901; doi:10.7554/eLife.16072)
Supplement: Supplementary file 2. — DOI: http://dx.doi.org/10.7554/eLife.16072.023 [file elife-16072-supp2.docx]

**Table S2: Comparison of UNC-75 targets identified in this study and those identified in RNAseq studies***

| UNC-75 CLIP-targets overlap with those in Kuroyanagi et al (2013) | UNC-75 CLIP-targets overlap with those in Norris et al (2014) | UNC-75 CLIP-targets overlap with those shown significant effects in PLM regrowth (Chen et al, 2011) | Common UNC-75 targets between Kuroyanagi et al (2013) and Norris et al (2014) |
| --- | --- | --- | --- |
| *tom-1* | *tom-1* | *hrp-1* | *tom-1* |
| *nrx-1* | *nrx-1* | *jip-1* | *nrx-1* |
| *unc-32* | *pat-12* | *nkb-1* | *clip-1* |
| *cdgs-1* | *bli-4* | *rig-3* |  |
| *ret-1* | *unc-49* | *unc-104* |  |
| *unc-16* | *unc-64* | *unc-115* |  |
| *unc-75* | *mrp-1* | *unc-32* |  |
| *daf-3* | *cca-1* | *unc-41* |  |
| *vab-10* | *pkg-2* | *unc-44* |  |
| *larp-1* |  | *unc-75* |  |
| *tag-60 (nrfl-1)* |  | *daf-3* |  |
|  |  | *ddr-2* |  |
|  |  | *gar-3* |  |
|  |  | *pqn-55* |  |
|  |  | *rig-4* |  |
|  |  | *unc-64* |  |
|  |  | *unc-70* |  |
|  |  | *hrp-1* |  |
|  |  | *jip-1* |  |
|  |  | *nkb-1* |  |
|  |  | *rig-3* |  |
|  |  | *unc-104* |  |
|  |  | *unc-115* |  |
|  |  | *unc-32* |  |

*Gene comparison used supplemental data from:

Kuroyanagi, H., Watanabe, Y., Suzuki, Y. & Hagiwara, M. Position-dependent and neuron-specific splicing regulation by the CELF family RNA-binding protein UNC-75 in *Caenorhabditis elegans*. *Nucleic acids research* **41**, 4015-4025 (2013).

Norris, A.D.*, et al.* A pair of RNA-binding proteins controls networks of splicing events contributing to specialization of neural cell types. *Molecular Cell* **54**, 946-959 (2014)

Chen, L.*, et al.* Axon regeneration pathways identified by systematic genetic screening in *C. elegans*. *Neuron* **71**, 1043-1057 (2011).
