# Supplementary material for: CELF RNA binding proteins promote axon regeneration in C. elegans and mammals through alternative splicing of Syntaxins
Source: eLife. 2016 Jun 2;5:e16072. doi: 10.7554/eLife.16072 (PMC4946901; doi:10.7554/eLife.16072)
Supplement: Supplementary file 3. — DOI: http://dx.doi.org/10.7554/eLife.16072.024 [file elife-16072-supp3.docx]

**Table S3. Summary of expression patterns of mouse CELF transcripts based on Allen Brain Atlas.**

| CELF members  (Other names) | CELF1  CUGBP1  BRUNOL2 | CELF2  CUGBP2 ETR-3 | CELF3  TNRC4 BRUNOL1 | CELF4  BRUNOL4 | CELF5  BRUNOL5 | CELF6  BRUNOL6 |
| --- | --- | --- | --- | --- | --- | --- |
| Brain (day4) | N/A | N/A | N/A | N/A | N/A | N/A |
| Brain (day56) | Weak expression | Widely and highly expressed in brain | Widely expressed in brain | Widely and highly expressed in brain | Weak expression | Weak expression |
| Spinal cord (day4) | Weak expression | Laminae 1-3 & Grey matter | Laminae 1-3 & Intermediolateral column & Grey matter | No/weak expression | Weak expression | Laminae 4-6 & Intermediolateral column & Grey matter |
| Spinal cord (day 56) | Weak expression | Laminae 1-3 & Grey matter | Laminae 1-3 & Intermediolateral column & Grey matter | Laminae 1-3 & 4-6  & Grey matter | Weak expression | Laminae 4-6 & Intermediolateral column & Grey matter |
