# Supplementary material for: CELF RNA binding proteins promote axon regeneration in C. elegans and mammals through alternative splicing of Syntaxins
Source: eLife. 2016 Jun 2;5:e16072. doi: 10.7554/eLife.16072 (PMC4946901; doi:10.7554/eLife.16072)
Supplement: Supplementary file 5. — DOI: http://dx.doi.org/10.7554/eLife.16072.026 [file elife-16072-supp5.docx]

**Table S5. *C. elegans* strains, transgenes, and clones**

| **Strains** | **Genotype** | **New DNA construct** | **pCZGY#** |
| --- | --- | --- | --- |
| CZ10969 | *Pmec-7-GFP(muIs32) II* |  |  |
| CZ11007 | *unc-75(e950) I; Pmec-7-GFP(muIs32) II* |  |  |
| CZ14053 | *unc-75(md1309) I; Pmec-7-GFP(muIs32) II* |  |  |
| CZ14054 | *unc-75(md1344) I; Pmec-7-GFP(muIs32) II* |  |  |
| CZ15531 | *unc-75(e950) I; Pmec-7-GFP(muIs32) II;Punc-75-UNC-75(juEx4320)* | P*unc-75*-UNC-75 (mini) | 2978 |
| CZ12797 | *unc-75(e950) I; Pmec-7-GFP(muIs32) II;Pmec-4-UNC-75(juEx3031)* | P*mec-4*-UNC-75 | 2981 |
| CZ15899 | *unc-75(e950) I; Pmec-7-GFP(muIs32) II Prgef-1-GFP::UNC-75(juSi76) II* | P*rgef-1*-GFP::UNC-75 (mini mosSCI) | 2983 |
| CZ15598 | *Pmec-4-GFP::UNC-75 (cDNA) (juEx4352)* | P*mec-4*-GFP::UNC-75 (cDNA) | 2984 |
| CZ15519 | *Pmec-4-GFP::UNC-75 (cDNA 1-472aa) juEx4308)* | P*mec-4*-GFP::UNC-75 (cDNA 1-472aa) | 2986 |
| CZ15527 | *unc-75(e950) I; muIs32 II; Punc-75- UNC-75(cDNA) (juEx4316)* | P*unc-75*-UNC-75(cDNA) | 2987 |
| CZ24173 | *unc-75(e950) I; muIs32 II; Punc-75-UNC-75(cDNA∆NLS) (juEx7397)* | Punc-75-UNC-75(cDNA∆NLS) | 2989 |
| CZ13825 | *unc-75(e950) I; Punc-25-GFP(juIs76) II* |  |  |
| CZ16621 | *unc-75(e950)I; Prgef-1-GFP::UNC-75(juSi76) II Punc-25-GFP(juIs76) II* |  |  |
| CZ19235 | *dlk-1(tm4024) I unc-75(e950) I ; Pmec-7-GFP(muIs32) II* |  |  |
| CZ13219 | *Pmec-4-GFP(zdIs5) I; Pmec-4-GFP::DLK-1L(juEx2529)* |  |  |
| CZ13627 | *unc-75(e950) I ; Pmec-7-GFP(muIs32) II; Pmec-4-GFP::DLK-1L(juEx2529)* |  |  |
| CZ13626 | *unc-75(e950) I ; mec-7-GFP(muIs32) II ; efa-6(tm3124) IV* |  |  |
| CZ9296 | *exc-7(rh252) II Pmec-7-GFP(muIs32) II* |  |  |
| CZ13998 | *unc-75(e950) I ; mec-7-GFP(muIs32) II exc-7(rh252) II* |  |  |
| CZ14662 | *unc-75(e950) I ; Prgef-1-FLAG::UNC-75(juIs369)* | P*rgef-1*-  BLRP::FLAG::UNC-75(cDNA) | 2991 |
| CZ17359 | *Pmec-7-GFP(muIs32) II; unc-64(md130) III* |  |  |
| CZ12095 | *dlk-1(ju476) I; Pmec-7-GFP(muIs32) II* |  |  |
| CZ17746 | *Punc-64::unc-64a(genomic)::gfp(juEx5292)* |  | (pTX34) |
| CZ17747 | *Punc-64::unc-64b(genomic)::gfp(juEx5293)* |  | (pTX37) |
| CZ17748 | *Punc-64::unc-64b (genomicΔ38nt)::gfp(juEx5294)* | P*unc-64*::unc-64b (genomicΔ38nt)::gfp | pCZ938 |
| CZ18891 | *Prgef-1-unc-64e7-8arfp-8b-gfp(juEx5668)* | P*rgef-1*-unc-64e7-8arfp-8b-gfp | 2993 |
| CZ18892 | *unc-75(e950) I ; Prgef-1-unc-64e7-8arfp-8b-gfp(juEx5668)* |  |  |
| CZ17333 | *unc-64(md130) III ; mec-7-GFP(muIs32) II ; Prgef-1-unc-64a(juEx5155)* | P*rgef-1*-unc-64a | 2996 |
| CZ17337 | *unc-64(md130) III ; mec-7-GFP(muIs32) II ; Prgef-1-unc-64b(juEx5159)* | P*rgef-1*-unc-64b | 2997 |
| CZ18888 | *unc-64(md130) III ; mec-7-GFP(muIs32) II ; Prgef-1-unc-64ΔTM(juEx5665)* | P*rgef-1*-unc-64 ΔTM | 2999 |
| CZ17335 | *mec-7-GFP(muIs32) II ; Prgef-1-unc-64a(juEx5157)* |  |  |
| CZ17339 | *mec-7-GFP(muIs32) II ; Prgef-1-unc-64b(juEx5161)* |  |  |
| CZ18197 | *mec-7-GFP(muIs32) II ; unc-64(e246) III* |  |  |
| CZ18338 | *mec-7-GFP(muIs32) II ; unc-64(e246) III; Prgef-1-unc-64a* |  |  |
| CZ24076 | *mec-7-GFP(muIs32) II ; unc-64(e246) III; Prgef-1-unc-64b* |  |  |
| CZ19614 | *unc-64(js115) III ; mec-7-GFP(muIs32) II ; Punc-17::syx;Pglr-1::syx;Pacr-2::syx;Pmyo-2::gfp(oxEx705)* |  |  |
| CZ23920 | *unc-64(js115) III ; mec-7-GFP(muIs32) II ; juSi316 V* |  |  |
| CZ23925 | *unc-64(js115) III ; mec-7-GFP(muIs32) II ; juSi316 V; P7-nCre(juEx7297)* |  |  |
| CZ24075 | *mec-7-GFP(muIs32) II ; P7-nCre(juEx7297)* |  |  |
| CZ18899 | *unc-75(e950) I ; mec-7-GFP(muIs32) II ; Prgef-1-unc-64a(juEx5158)* |  |  |
| CZ18900 | *unc-75(e950) I ; mec-7-GFP(muIs32) II ; Prgef-1-unc-64b(juEx5161)* |  |  |
| CZ18884 | *unc-75(e950) I ; mec-7-GFP(muIs32) II ; Punc-64-unc-64(genomic)-gfp(juEx5661)* |  |  |
| CZ18894 | *unc-75(e950) I ; mec-7-GFP(muIs32) II ; Prgef-1-unc-64 ΔTM (juEx5671)* |  |  |
| CZ18339 | *mec-7-GFP(muIs32) II ; unc-75(e950) I ; Pmec-4-mc-mCELF2 (juEx5488)* | P*mec-4*-mc-mCELF2 | 3002 |
| CZ18341 | *mec-7-GFP(muIs32) II ; unc-75(e950) I ; Pmec-4-mc-mCELF4(juEx5490)* | P*mec-4*-mc-mCELF4 | 3003 |
