# Supplementary material for: CELF RNA binding proteins promote axon regeneration in C. elegans and mammals through alternative splicing of Syntaxins
Source: eLife. 2016 Jun 2;5:e16072. doi: 10.7554/eLife.16072 (PMC4946901; doi:10.7554/eLife.16072)
Supplement: Supplementary file 6. — DOI: http://dx.doi.org/10.7554/eLife.16072.027 [file elife-16072-supp6.docx]

**Table S6. Primers used for RT-qPCR**

| Primer position | Target mRNA | Primer sequence (5’-3’) |
| --- | --- | --- |
| dlk-1 E10-F | dlk-1 | CAGCCCGTCTTCTTTACACG |
| dlk-1 E11-R | dlk-1 | GAGCTGGTGAGGAGCATTTC |
| efa-6 E9-F | efa-6 | GGGTTGCCGAACTAAATGAA |
| efa-6 E10-R | efa-6 | GACCGTCGGAGTACGTTGAT |
| efa-6 E4-F | efa-6 | TCGTACAATTCGTCACCACAA |
| efa-6 E5-R | efa-6 | TGACATATCCTTTGCGTCCA |
| unc-64 E7-F | unc-64a/b | TGTTGATCGAGCAGTAGCTGA |
| unc-64 E8a-R | unc-64a | GAAGCCGATTAGGATGGTGA |
| unc-64 E8b-R | unc-64b | GGCCAGTGATGAGTATGACG |
| unc-64 E6-F | unc-64 total | ACGCTAGCCGATATTGAAGC |
| unc-64 E7-R | unc-64 total | GCGTGCTCCACATTGTACTC |
| Celf2 E11-12F | Celf2 | CACAAGCCCTGTGGCTGCTT |
| Celf2 E12-13R | Celf2 | TTCAGAGCCGCCATACCTGC |
| Celf2 E13-14F | Celf2 | AGCCAGAAGGAAGGTCCAGAG |
| Celf2 E14R | Celf2 | GCACTTGCTCAGATTGGTCTGTTTG |
| Celf4 E9-10-F | Celf4 | GCAGGACCTGCCTACCCTGC |
| Celf4 E11-R | Celf4 | GGGCAGATGGTAGATGAGCA |
| stx1a E8-F | stx1a | CCCTCAGTGAGATCGAGACC |
| stx1a E9-R | stx1a | CGTGCTCCACATTGTACTCG |
| stx1b E9-F | stx1b | GGTGAGATGATCGACCGAAT |
| stx1b E10-R | stx1b | CAATGGATGACGCCAAGAC |
| stx2 E9-F | stx2-001/2 | TGGTCAACAACATCGAGAGAA |
| stx2-001 E10-R | stx2-001 | GCCAATGATTAGAGCCAGGA |
| stx2-002 E10-R | stx2-002 | ATGACAATGCTGTTGCGAGA |
| stx3 E8-F | stx3-201/2 | CTCCCAGATTTCCAAGCAAG |
| stx3-201 E9-R | stx3-201 | CCACTGACTGGTCCATGTTG |
| stx3-202 E9-R | stx3-202 | CCTTCTCCACATGGTCCACT |
| stx5a E7-8-F | stx5a | AACAACCTTGGAGGTGGTCC |
| stx5a E9-R | stx5a | CCCGACTCTGGATGTAGGAA |
| stx13 E6-7-F | stx13 | CAGCTGGAGGCTGACATTTTG |
| stx13 E8-R | stx13 | AGTGGCTCTTTCCACATGCA |
| stx16-001 E1-2F | stx16-001 | CATTGCTGCGGAGCTGGACG |
| stx16-201 E1-2F | stx16-201 | TTGCTGCGGAGTGTGATACAG |
| stx16 E3-4R | stx16-001/201 | ACTGTATTTCGTCCACGCCA |
| stx18 E6-7-F | stx18 | GCTGCCAGAAAAGCCTTTGG |
| stx18 E8-R | stx18 | CTTCGTCAAACAAGCTGTTCA |
